# A novel clinically relevant human fecal microbial transplantation model in humanized mice

**DOI:** 10.1128/spectrum.00436-24

**Published:** 2024-08-20

**Authors:** Shuai Yang, Linglin Tong, Xin Li, Yuchen Zhang, Hao Chen, Wei Zhang, He Zhang, Yang Chen, Renjin Chen

**Affiliations:** 1 College of Life Sciences, Xuzhou Medical University, Xuzhou, Jiangsu, China; 2 Department of Neurology, the Affiliated Hospital of Xuzhou Medical University, Xuzhou, Jiangsu, China; Ruijin Hospital, Shanghai Jiaotong University, Shanghai, China

**Keywords:** humanized mice, gut microbiota, immune system, fecal microbiota transplantation, colonization

## Abstract

The intact immune system of mice exhibits resistance to colonization by exogenous microorganisms, but the gut microbiota profiles of the humanized mice and the patterns of human fecal microbiota colonization remain unexplored. Humanized NCG (huNCG) mice were constructed by injected CD34 +stem cells. 16S rRNA sequencing and fecal microbiota transplantation (FMT) technologies were used to detect the differences in microbiota and selective colonization ability for exogenous community colonization among three mice cohorts (C57BL/6J, NCG, and huNCG). Flow cytometry analysis showed that all huNCG mice had over 25% hCD45 +in peripheral blood. 16S rRNA gene sequence analysis showed that compared with NCG mice, the gut microbiota of huNCG mice were significantly altered. After FMT, the principal coordinates analysis (PCoA) showed that the gut microbial composition of huNCG mice (huNCG-D9) was similar to that of donors. The relative abundance of Firmicutes and Bacteroidetes were significantly increased in huNCG mice compared to NCG mice. Further comparison of ASV sequences revealed that *Bacteroides plebeius*, *Bacteroides finegoldii*, *Escherichia fergusonii*, *Escherichia albertii*, *Klebsiella pneumoniae,* and *Klebsiella variicola* exhibited higher abundance and stability in huNCG mice after FMT. Furthermore, PICRUSt2 analysis showed that huNCG mice had significantly enhanced metabolism and immunity. This study demonstrated that humanized mice are more conducive to colonization within the human gut microbiota, which provides a good method for studying the association between human diseases and microbiota.

**IMPORTANCE:**

The gut microbiota and biomarkers of humanized mice are systematically revealed for the first time. The finding that human fecal microbiota colonize humanized mice more stably provides new insights into the study of interactions between immune responses and gut microbiota.

## INTRODUCTION

The gut microbiome consists of approximately 3 × 10^13^ bacteria (1) and performs various roles in the human host, including breaking down hard-to-absorb food, producing vitamins, protecting against harmful organisms, enhancing the intestinal barrier function, and controlling the immune system (2–7). Gut microbial colonization plays a mutually reinforcing role with the developmental processes of the immune system. Microbes are already existing during the prenatal phase, facilitating the growth and refinement of the immune system (8). The development of the immune system in the intestines depends critically on the microbiota in newborns’ gastrointestinal tracts (9). Studies on germ-free (GF) mice have also shown that the absence of gut microbiota leads to significant immune system deficiencies (10–12).

The immune response of conventional mice is different from that of humans, making it difficult to successfully apply preclinically validated immunotherapeutic regimens in the clinic (13–15). To address this limitation, researchers are employing humanized mice that possess a fully functional human immune system, including T and B lymphocytes as well as natural killer (NK) cells (16, 17). Currently, the utilization of humanized mice has become extensive in the assessment of the impacts of immunotherapy, exploration of tumor immune evasion mechanisms, and deciphering the immune cell’s antitumor mechanisms (18–21). Furthermore, the immune system regulates the balance of microorganisms and prevents infection, while also impacting the establishment of microbial communities (22–24). Additionally, it enables the examination of host–pathogen interactions as well as the interactions between the immune system and intestinal microbiota. Despite the extensive utilization of humanized mice in tumor immunology, less exploration of intestinal microbiota in these mice has been carried out. Antibiotic cocktail treatment is a commonly applied method for FMT, and it significantly reduces the abundance and diversity of gut microbes after 1 week, whereas lower gut microbial diversity and abundance in recipient mice can help the donor microbiota better colonize the gut microbiota (25, 26). Mice with the immune phenotype show resistance to the colonization of the human gut microbiota (27, 28), and it is unclear whether the humanized immune system would alter the colonization patterns of the human gut microbiota.

This study aimed to explore the rapid colonization of exogenous bacteria in the humanized mice and establish an ideal mouse model of human immune system–bacteria interactions. A humanized mouse model was constructed using uncultured fresh CD34^+^ hematopoietic stem cells (CD34^+^ HSCs) (29, 30). To simulate the colonization of exogenous microbials and elucidate the colonization pattern of intestinal microbiota in healthy individuals, human fecal microbiota was transplanted into three types of mice: C57BL/6 J mice with an intact immune system, NCG mice that are severely immunodeficient, and huNCG mice with a humanized immune system. The results of 16S rRNA gene sequencing indicate that the colonization efficiency and stability of *Bacteroides plebeius*, *Bacteroides finegoldii*, *Klebsiella pneumoniae*, *Klebsiella variicola*, and *Escherichia coli* strain are higher in humanized mice than in NCG mice. This can further the understanding of host immune responses and resistance to pathogens, evaluating the efficacy of therapeutic probiotics, and improve humanized mouse models associated with the human microbiota.

## RESULTS

### Construction of the humanized mouse model

**Fig 1:**
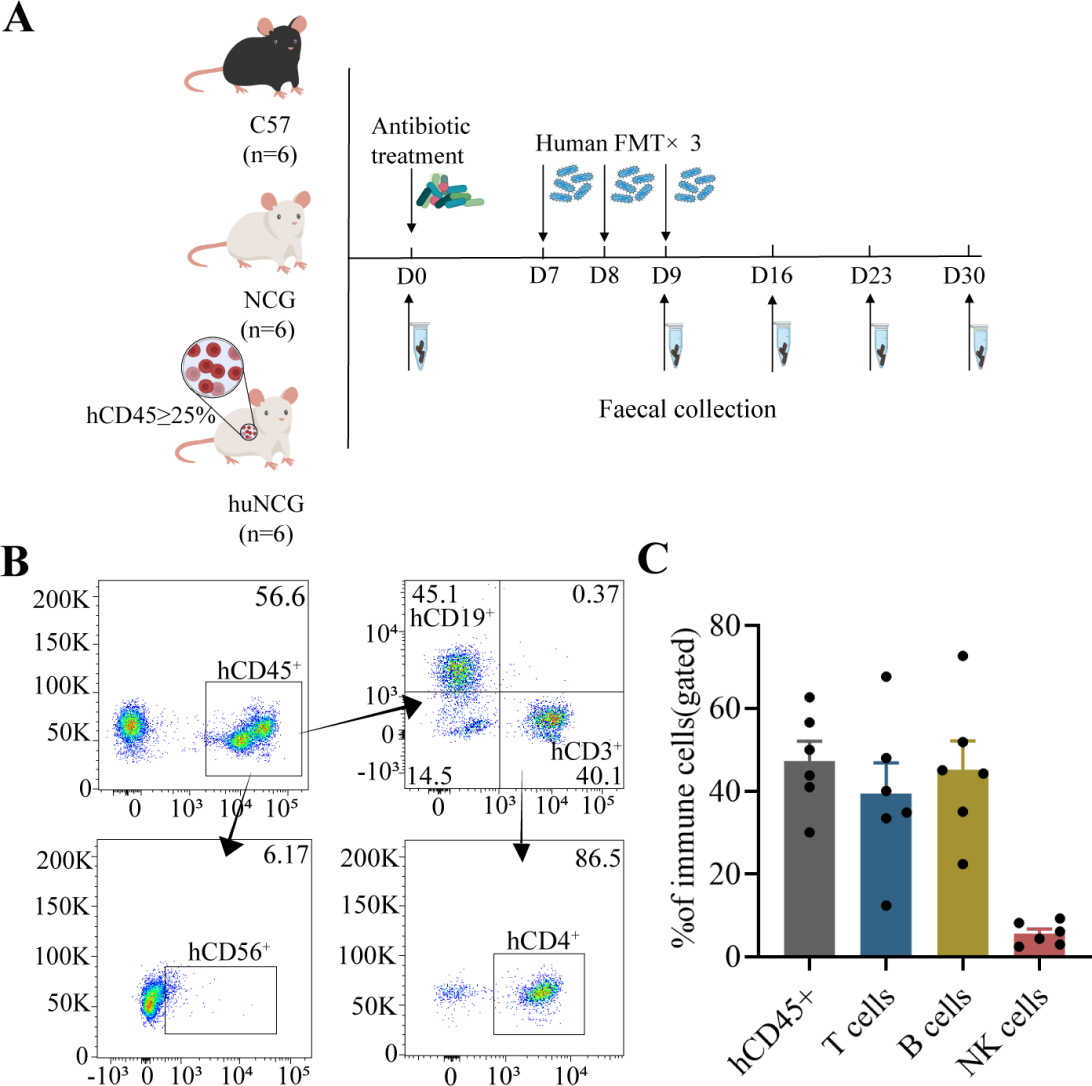
Establishing the humanized mice model by injecting CD34 +cells. (**A**) Schematic representation of the workflow to establish the different FMT models for evaluation of the colonization tendency in each group of mice. (**B**) Analysis of gating strategies for human immune cells in the peripheral blood of mice progressively gated human immune subpopulations, such as T cells, B cells, and NK cells. (**C**) After implanting cells, the immune profile of humanized mice in the peripheral blood (*n* = 6) was analyzed at 8 weeks.

According to the experimental design for human FMT (Fig. 1A), we generated humanized NCG mice by irradiating NCG mouse pups with 120 cGy and injecting CD34^+^ HSCs into livers. After 8 weeks of transplantation, flow cytometry analysis was performed following gating strategies (Fig. 1B). In the peripheral blood of humanized mice, the average percentage of hCD45^+^ cells was 47.4%. CD3^+^ T cells accounted for an average of 39.4% of total human leukocytes in the peripheral blood, with CD4^+^ T helper cells representing an average of 80.9% of the T cell population. CD19^+^ B cells accounted for an average of 45.3% of total human leukocytes in the peripheral blood, while CD56^+^ NK cells accounted for an average of 5.61% (Fig. 1C).

### Alterations in the gut microbiota of humanized mice

**Fig 2:**
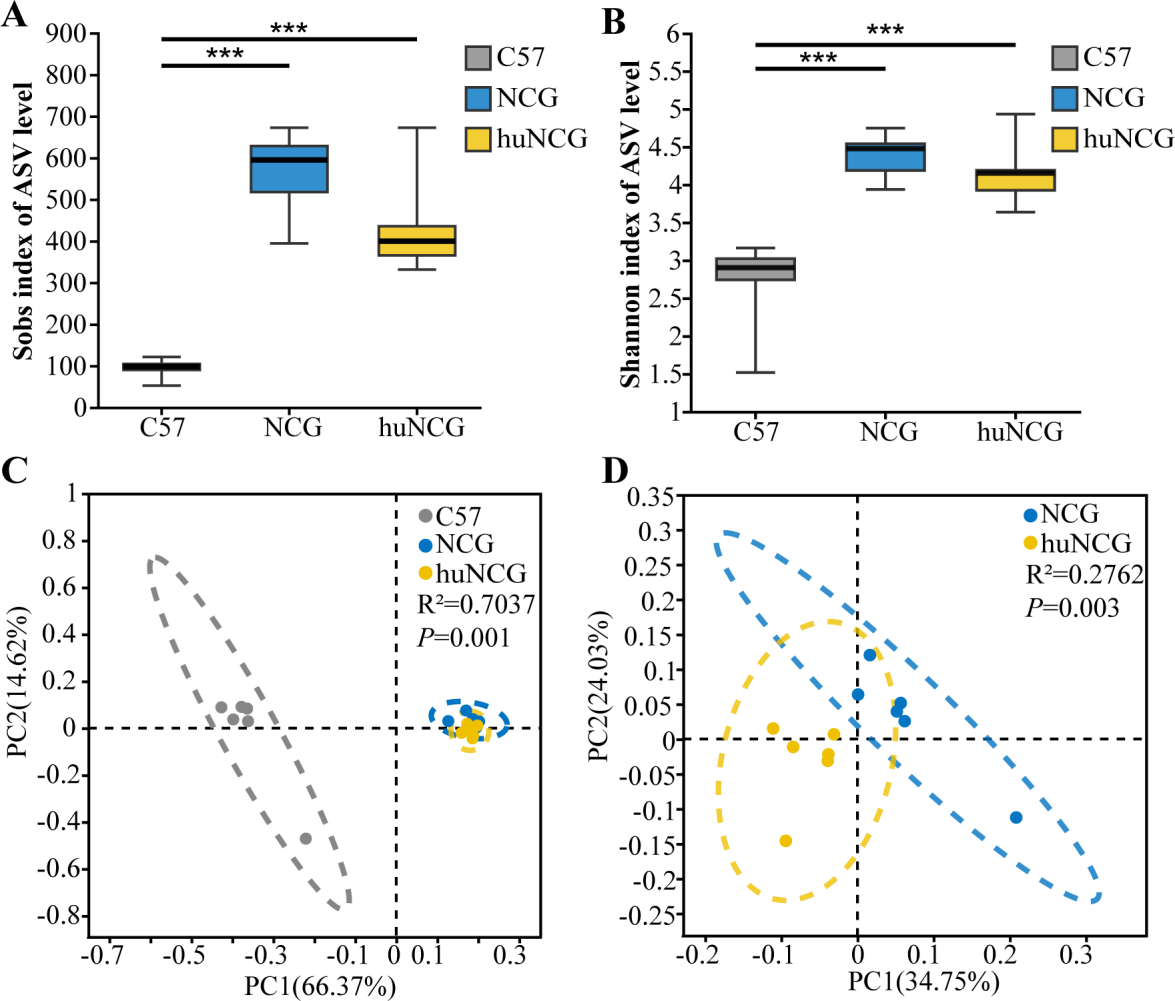
Reconstruction of the human immune system results in modification of the gut microbiota in mice. The impact of the human immune system on α-diversity was assessed by utilizing the (**A**) Sobs index and (**B**) Shannon index. (**C, D**) β-diversity is determined by utilizing unweighted UniFrac distances and conducting principal coordinate analysis (PCoA). ^*^P <0.05, ^**^P <0.01, and ^***^P<0.001.

We further examine the gut microbiota of each group prior to antibiotic treatment to better understand how the human immune system affects the mouse gut microbiota. First, the dilution curve constructed based on the Shannon index and Sobs index showed that the dilution curves of each sample smoothed out, and the number of sequences was sufficient to cover the microbial communities in all samples (Fig. S1A and B). The α-diversity index was calculated using the Sobs index and Shannon index to measure the richness and diversity. While there was no difference between NCG mice and huNCG mice, NCG mice and huNCG mice had significantly enhanced Sobs index and Shannon index when compared to C57BL/6 J mice (Fig. 2A and B). The immune deficiency and transplantation of the human immune system contributed to the increase in α-diversity. PCoA was used to determine the diversity in order to evaluate the differences in microbial composition across groups. Between C57BL/6 J mice, NCG mice, and huNCG mice, there were significant differences in the composition of the gut microbiota (*P* < 0.01), (Fig. 2C). While the overall groups showed a similar gut microbiota composition in both NCG mice and huNCG mice, there were notable distinctions in the gut microbiota composition between these two groups (*P* < 0.01) (Fig. 2D).

### Identification of special biomarkers in the gut microbiota of humanized mice

**Fig 3:**
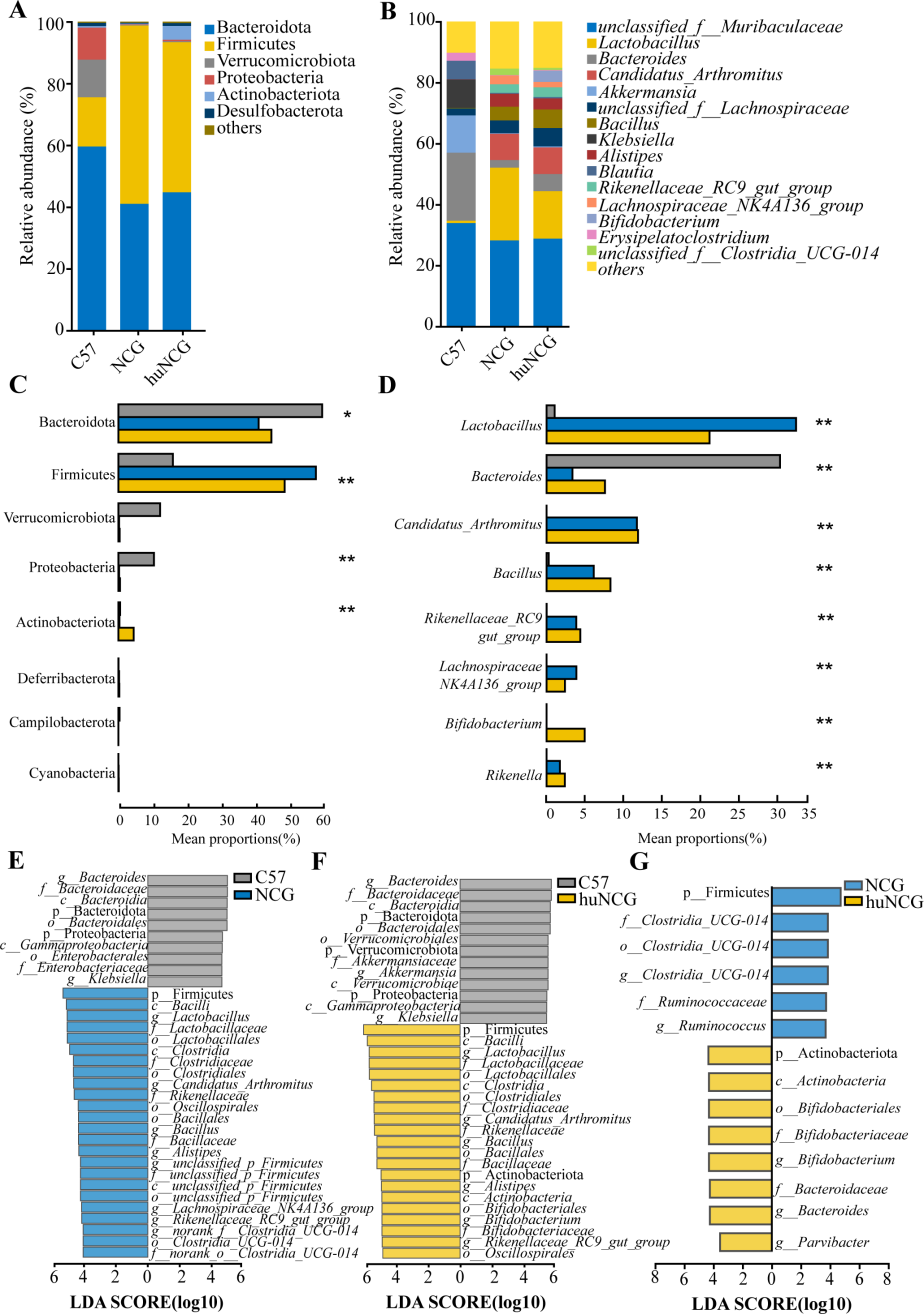
Reconstruction of the human immune system alters the composition of the gut microbiota. (A) The phylum-level relative abundance of all gut microbiota for each group. (**B**) Differences in the relative abundance of different groups at the phylum level. (**C**) Relative abundance of each group’s gut microbiota in the top 15 genera at the genus level. (**D**) Differences in the relative abundance of different groups at the genus level. (**E–G**) The LDA score signifies the extent of variation among the three groups. A bar chart displayed horizontally indicates the taxa that discriminate. A threshold value of 4.0 was used for comparison of C57BL/6J with NCG and C57BL/6J with huNCG; a threshold value of 3 was used for comparison of NCG with huNCG. Significant discriminant taxa of the C57BL/6J, NGC, and huNCG are represented by gray, blue, and yellow, respectively. Data are expressed as mean ± SEM (*n* = 6). ^*^P <0.05; ^**^P <0.01, and ^***^P <0.001.

At the phylum and genus levels, the composition of the gut microbiota was further examined. At the phylum level, huNCG mice had a considerably greater relative abundance of Actinobacteria than NCG mice and huNCG mice (*P* < 0.01), which was different from the observations in the other two groups. Firmicutes and Proteobacteria had a considerably higher relative abundance in C57BL/6 J mice (*P* < 0.01), while the relative abundance of Bacteroidetes was significantly lower (*P* < 0.05) (Fig. 3A and C). At the genus level, compared to C57BL/6 J mice, huNCG mice and NCG mice showed significantly increased relative abundance of *Lactobacillus*, *Candidatus_Arthromitus*, *Bacillus*, *Alistipes*, *Rikenellaceae_RC9_group*, *Lachnospiraceae_NK4A136_group*, and *Rikenella* (*P* < 0.01), while the relative abundance of *Bacteroides* was significantly lower (*P* < 0.01). It should be noted that *Bifidobacterium* was only present in huNCG mice and showed a significantly higher relative abundance (*P* < 0.01) compared to the other two groups (Fig. 3B and D).

In addition, the significant differences in bacterial communities among C57BL/6J, NCG, and huNCG mice were further analyzed using linear discriminant analysis (L D A) effect size method (LEfSe) at the phylum to genus levels. The results showed differential relative abundances of taxa at different levels in each group. Bacteroides, Proteobacteria, Gammaproteobacteria, and Enterobacteriaceae were identified as biomarkers for C57BL/6 J mice. Firmicutes, Clostridiaceae, Lactobacillus, Rikenellaceae, Bacillus, and Candidatus_Arthromitus were identified as biomarkers for NCG mice (Fig. 3E). Compared to C57BL/6 J mice, Firmicutes, Bacilli, Lactobacillus, Rikenellaceae, Candidatus_Arthromitus, Rikenellaceae, *Alistipes*, and *Bifidobacterium* were identified as biomarkers for huNCG mice, which were almost consistent with the biomarkers for NCG mice (Fig. 3F). However, compared to NCG mice, Actinobacteriota, *Bifidobacterium*, *Bacteroides*, and *Parvibacter* were identified as biomarkers for huNCG mice (Fig. 3G).

### Humanized mice had a similar gut microbiota with the donor after FMT

To further investigate the colonization and resistance to human microbiota in huNCG mice with a human immune system, we collected fecal samples for 16S rRNA gene sequencing after human FMT and analyzed the similarity between the gut microbiota composition of each group of mice and the human donor microbiota.

**Fig 4:**
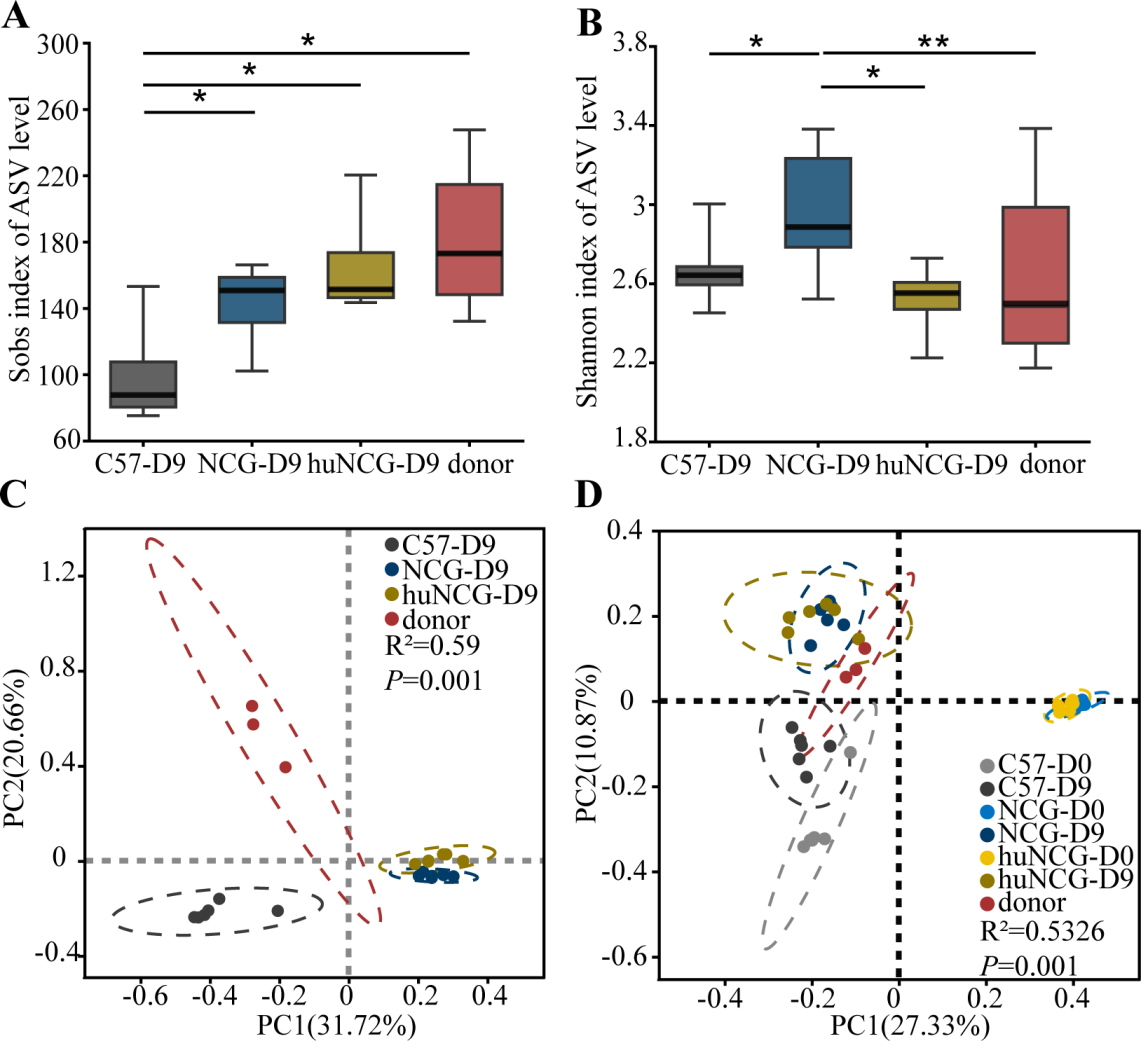
Influence of the immune system on gut microbiota after FMT. The effect of the immune system on α-diversity was determined using the Sobs index (**A**) and the Shannon index (**B**). (**C, D**) Determination of β-diversity using unweighted UniFrac distances based on principal coordinate analysis. Data are expressed as mean ± SEM (*n* = 6). ^*^P<0.05, ^**^P<0.01, and ^***^P<0.001.

To reveal the richness and diversity, the Sobs index and Shannon index of α-diversity were calculated. Compared with C57BL/6 J mice after FMT (C57-D9), the Sobs index was significantly increased (*P* < 0.05) in NCG mice after human FMT (NCG-D9) and huNCG mice after FMT (huNCG-D9). However, there was no significant difference between the NCG-D9 group, the huNCG-D9 group, and the donor group (*P* > 0.05) (Fig. 4A). Compared to the C57-D9 group, the Shannon index significantly increased in the NCG-D9 group (*P* < 0.05), while it significantly decreased in the huNCG-D9 group compared to the NCG-D9 group (*P* < 0.05), but remained similar to the donor group (*P* > 0.05) (Fig. 4B). In terms of α-diversity, the gut microbiota of humanized mice after human FMT showed the highest similarity to the donor group. To assess the differences in the microbial composition between different groups, we performed principal coordinate analysis to calculate the β-diversity. The analysis of variance showed differences between the C57-D9 group, NCG-D9 group, huNCG-D9 group, and the donor group (*P* < 0.01) (Fig. 4C). Moreover, compared to the gut microbiota composition of C57BL/6 J mice after human FMT (C57-D0) and NCG mice after human FMT (NCG-D0), the gut microbiota composition of huNCG mice after human FMT (huNCG-D0) had significantly changed (*P* < 0.01) and became similar to that of the donor group (Fig. 4D).

### Effect of the human immune system on the colonization of human fecal microbes

**Fig 5:**
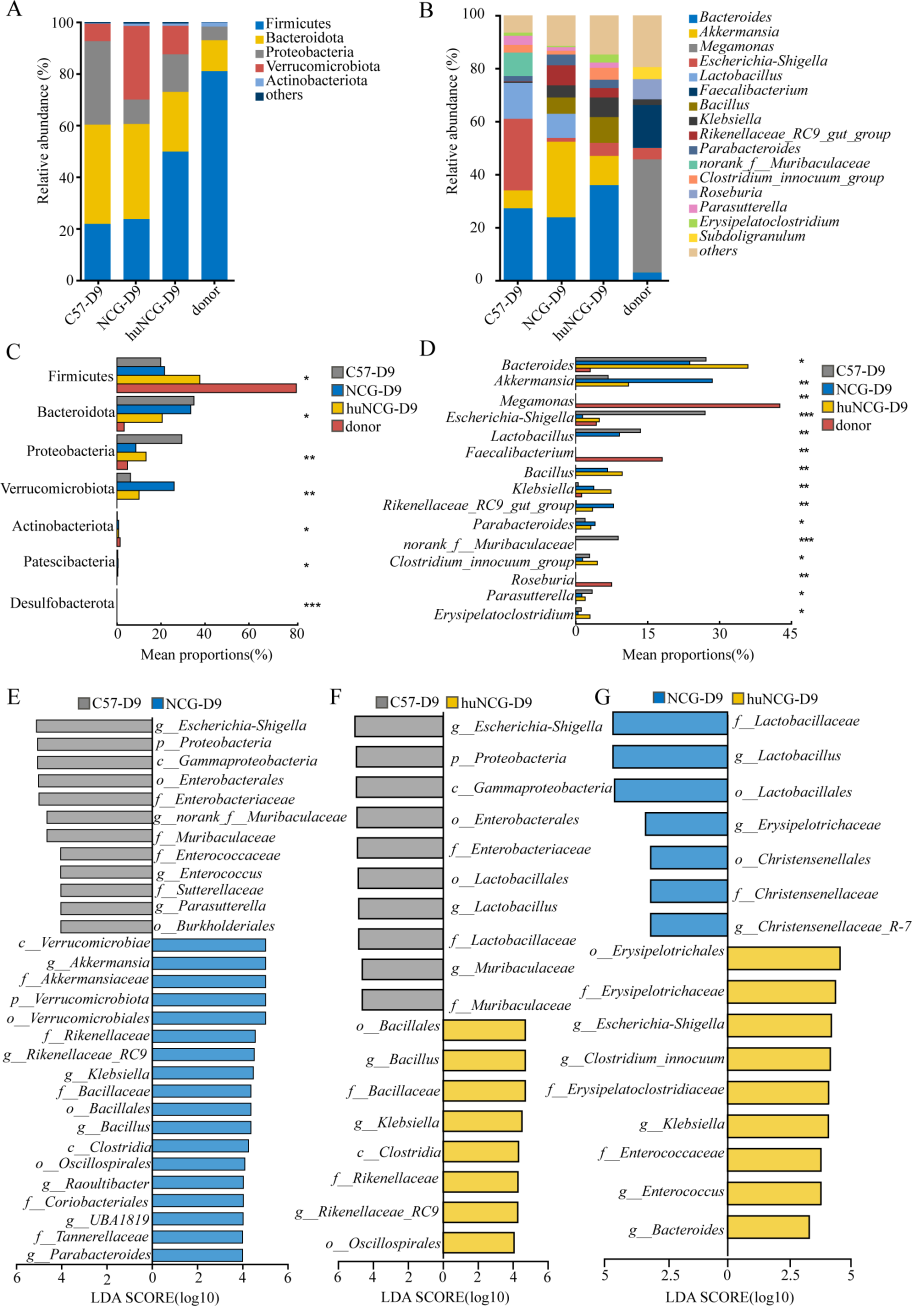
The immune system influenced the colonization efficiency of human fecal microbes. (**A**) The phylum-level relative abundance of all gut microbiota for each group. (**B**) Relative abundance of each group’s gut microbiota in the top 15 genera at the genus level. (**C**) Differences in the relative abundance of different groups at the phylum level. (**D**) Differences in the relative abundance of different groups at the genus level. (**E–G**) The LDA score signifies the extent of variation among the three groups. A bar chart displayed horizontally indicates the taxa that discriminate. A threshold value of 4.0 was used for comparison of C57 with NCG and C57 with huNCG; a threshold value of 3.0 was used for comparison of NCG with huNCG. Significant discriminant taxa of the C57, NGC, and huNCG are represented by gray, blue, and yellow, respectively. Data are expressed as mean ± SEM (*n* = 6). ^*^P <0.05; ^**^P <0.01, and ^***^P <0.001.

At the phylum and genus levels, the composition of the gut microbiota was further examined. At the phylum level, huNCG mice had a considerably higher relative abundance of Firmicutes than NCG mice (*P* < 0.05) (Fig. 5A and C). At the genus level, compared to NCG mice, huNCG mice showed a significantly increased relative abundance of *Bacteroides*, *Escherichia-Shigella*, *Bacillus*, *Klebsiella*, *Clostridium innocuum*, and *Erysipelatoclostridium* (*P* < 0.05), while the relative abundance of *Akkermansia*, *Lactobacillus*, and *Rikenellaceae RC9* was significantly lower (*P* < 0.05) (Fig. 5B and D).

In addition, the significant differences in bacterial communities among C57BL/6J, NCG, and huNCG mice were further analyzed using the linear discriminant analysis (L D A) effect size (LEfSe) method at the phylum to genus levels. The results showed differential relative abundances of taxa at different levels in each group. *Escherichia-Shigella*, *Proteobacteria*, *Gammaproteobacteria*, and Enterobacteriaceae were identified as biomarkers for C57BL/6 J mice. *Verrucomicrobiae*, Clostridiaceae, *Lactobacillus*, Rikenellaceae, *Bacillus*, and *Candidatus_Arthromitus* were identified as biomarkers for NCG mice (Fig. 5E). Compared to C57BL/6 J mice, *Bacillales*, *Bacilli*, *Klebsiella*, *Clostridia*, Rikenellaceae, Rikenellaceae RC9, and Oscillospirales were identified as biomarkers for huNCG mice, which were almost consistent with the biomarkers for NCG mice (Fig. 5F). However, compared to NCG mice, Erysipelotrichales, Erysipelotrichaceae, *Escherichia Shigella*, *Klebsiella*, Enterococcaceae, *Enterococcus*, and *Bacteroides* were identified as biomarkers for huNCG mice (Fig. 5G).

### Several species exhibited a higher colonization ability with the human immune system

**Fig 6:**
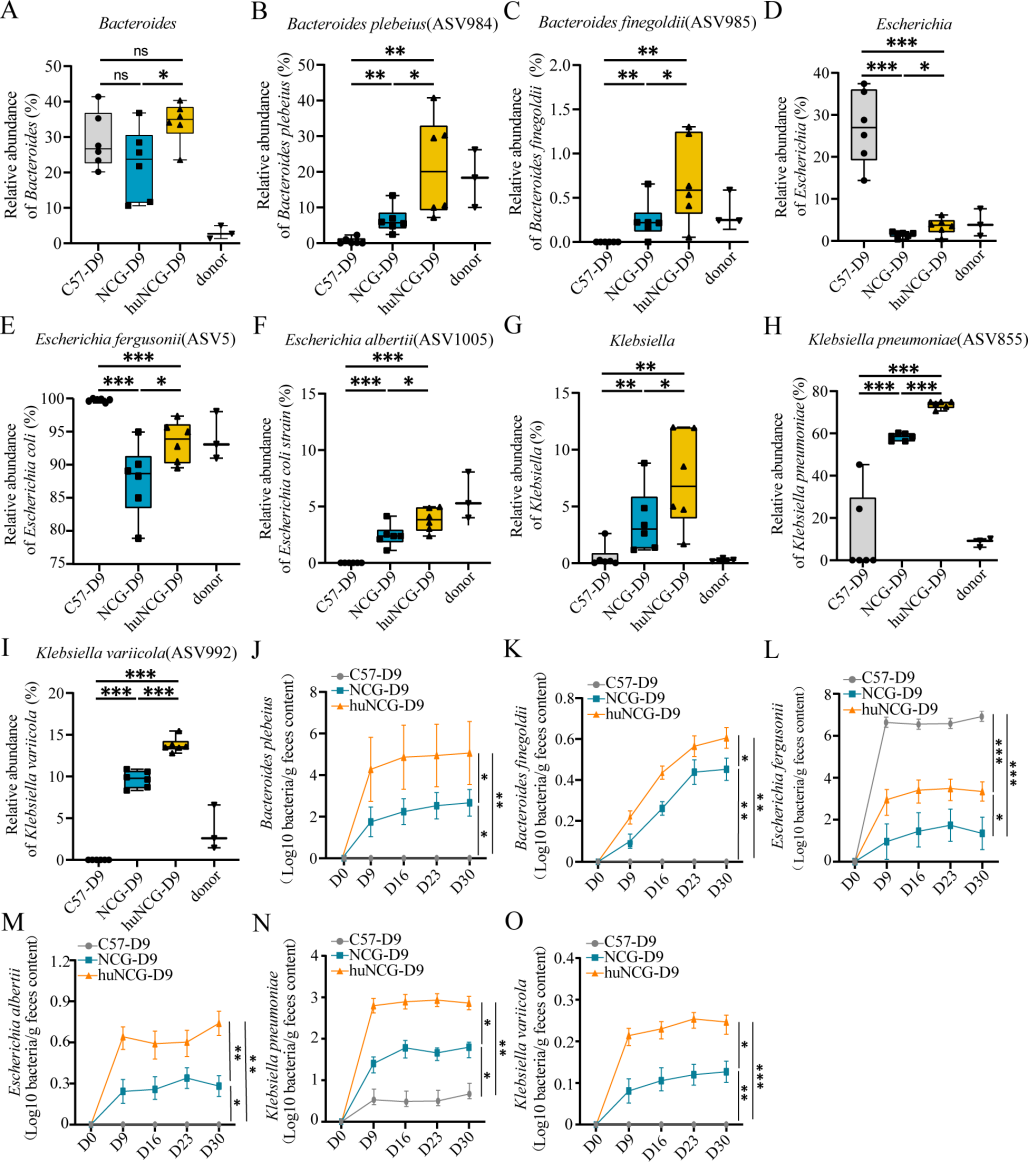
Relative abundances of some fecal microbes that colonized different immune systems. (**A, D, G**) *Bacteroides*, *Escherichia*, and *Klebsiella* in human donors and elevated in abundance after human FMT in the huNCG group. (**B, C, E, F, H, I**) Six species with a significant increase in abundance in three genera in huNCG mice. (**J–O**) The abundance changes of six species in fecal samples were quantitatively assessed over the subsequent 3 weeks. ^*^P<0.05, ^**^P<0.01, and ^***^P<0.001.

To identify the bacterial species that colonize the human fecal microbiota in humanized mice, the composition of the gut microbiota was further analyzed at the genus level. *Bacteroides*, *Escherichia*, and *Klebsiella* (Fig. 6A, D and G) were present in the donor group and were significantly increased in the huNCG-D9 compared to NCG-D9 (*P* < 0.05). In addition, six strains were obtained by aligning the amplicon sequence variant (ASV) 16S rRNA gene sequence using the Basic Local Alignment Search Tool (BLAST) tool. Phylogenetic trees constructed by the maximum likelihood method showed that ASV984 had a close genetic relationship with *Bacteroides plebeius*; ASV985 was closely related to *Bacteroides finegoldii* (Fig. S2A); there was a close genetic relationship between ASV5 and *Escherichia fergusonii*; ASV1005 was closely related to *Escherichia albertii* (Fig. S2B); ASV855 was closely related to *Klebsiella pneumoniae*; and ASV992 had a close genetic relationship with *Klebsiella variabilis* (Fig. S2C). The specific sequences of six ASVs have been provided (Table S1). Significant differences in abundance were identified in the huNCG-D9 compared to NCG-D9, including *Bacteroides plebeius* (*P* < 0.05, Fig. 6B), *Bacteroides finegoldii* (*P* < 0.05, Fig. 6C), *Escherichia fergusonii* (*P* < 0.05, Fig. 6E), *Escherichia albertii* (*P* < 0.05, Fig. 6F), *Klebsiella pneumoniae* (*P* < 0.001, Fig. 6H), and *Klebsiella variicola* (*P* < 0.001, Fig. 6I), indicating a better colonization of human fecal microbiota in humanized mice. Real-time PCR analysis revealed that *Bacteroides plebeius*, *Bacteroides finegoldii*, *Escherichia fergusonii*, *Escherichia albertii*, *Klebsiella pneumoniae,* and *Klebsiella variicola* (Fig. 6J through O) in huNCG mice could establish stable colonization after 3 weeks, with a significant difference in the proportions of these bacteria in the fecal microbiota compared to NCG mice.

### Functional pathways of gut microbiota in the human immune system resembling with the donor

**Fig 7:**
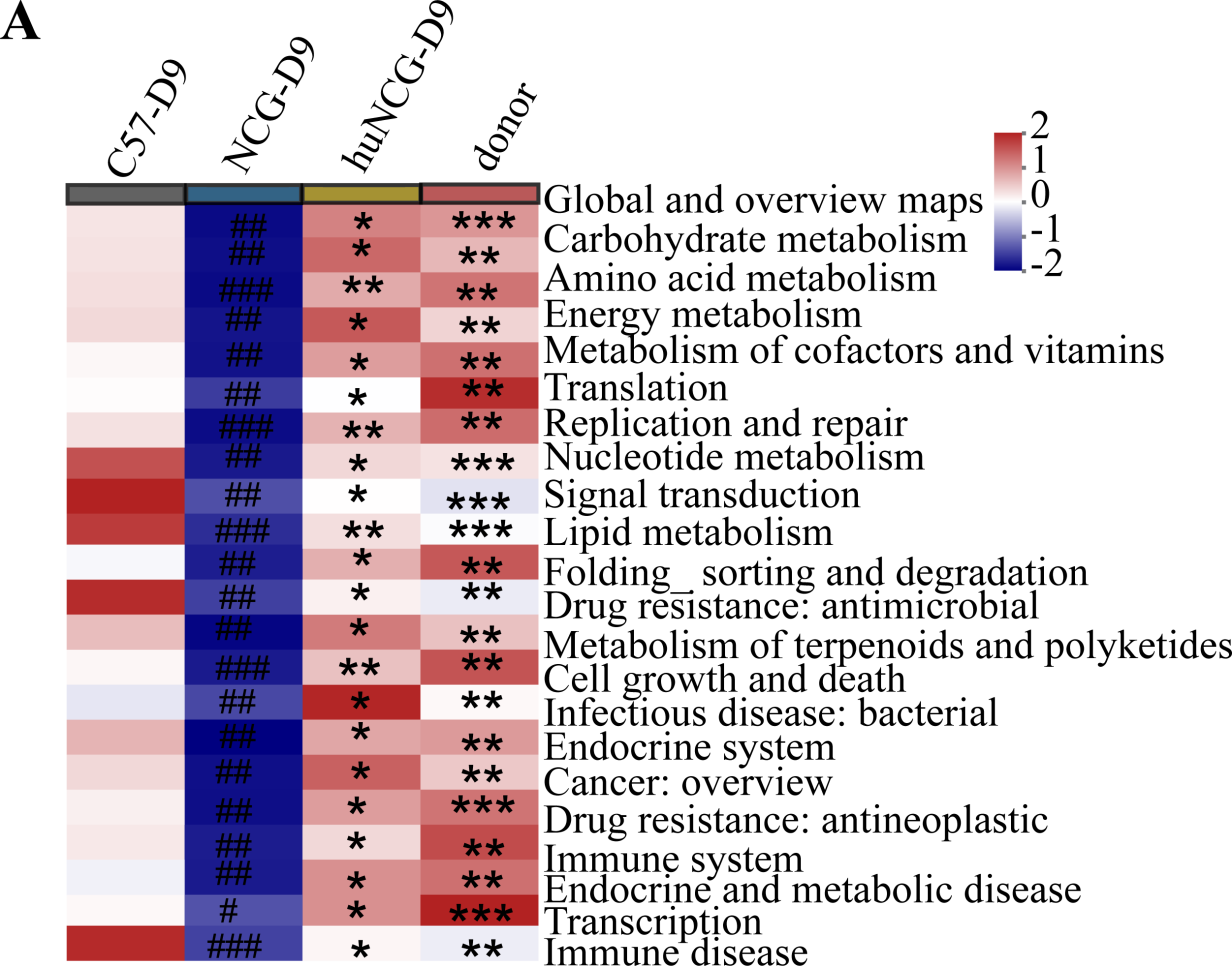
Prediction of better gut microbiota function in humanized mice based on the Kyoto Encyclopedia of Genes and Genomes (KEGG) database. (**A**) Humanized mice have similar gut microbiota function to donors after human FMT. ^＃^*P* < 0.05, ^＃＃^*P* < 0.01, and ^＃＃＃^*P* < 0.001 versus C57BL/6J; ^*^P < 0.05; ^**^P < 0.01, and ^***^P < 0.001 versus NCG.

KEGG functional prediction showed significant differences in the immune system and pathogenic bacterial function of huNCG mice after human FMT due to differences in immunophenotypes (*P* < 0.05) (Fig. 7A). The data showed that the gut microbiota function in huNCG mice was significantly positively correlated (*P* < 0.05). The functional profile of the gut microbiota of huNCG mice was more like that of human donors (*P* > 0.05).

## DISCUSSION

The humanized mouse models for the immune system have been widely used in research on autoimmune diseases, tumor immunotherapy, and pathogen infections, providing a more direct and reliable model for drug development and treatment strategy evaluation (19, 31, 32), which are important preclinical tools for biomedical research. The main humanized mice are constructed by engrafting human CD34 +stem cells in newborn or adult immunodeficient mice (33). The modes are used to study human-adapted pathogens like HIV, Ebola virus, dengue virus, *Staphylococcus aureus*, and cancer biology (34–36). There are many differences in the innate immune response between humans and mice (37). The immune system can maintain its balance and health by regulating the composition and function of the microbiota (22). Previous studies have demonstrated the interaction between the immune system and the microbiota, involving innate immune resistance exhibited by exogenous microbiota, and humanized mice are more suitable for studying gut microbiome–immune interaction (38, 39). However, there is a lack of further insights into the changes in the microbiota composition of humanized mice and the specific microorganisms that colonize rapidly and stably after exogenous microbial transplantation. In this study, the microbiota differences were characterized in NCG mice compared with huNCG mice, prior to human fecal microbiota transplantation. In addition, *Bacteroides plebeius*, *Bacteroides finegoldii*, *Klebsiella pneumoniae*, and *Klebsiella variicola* rapidly and persistently colonized humanized mice after FMT. This suggests that humanized mice can serve as an ideal animal model to study the interaction between the immune system and the gut microbiota in pathogen-induced diseases.

In the study, the humanized mice were successfully constructed with the human immune system. The percentage of human CD45 +cells was 47.4%, with percentage of T cells, B cells, and NK cells at 39.4%, 45.3%, and 5.61%, respectively, which were similar to the results observed in previous studies (40, 41). To explore the changes in the gut microbiota of huNCG mice, the difference of the intestinal flora of C57BL/6J, NCG, and huNCG mice was characterized. There are significant differences among the gut microbiota of NCG mice, huNCG, and C57BL/6 J mice. Also, huNCG mice and NCG mice have more similar gut microbiota. Therefore, the immunodeficiency may reshape the native microbiome, increasing the host’s susceptibility to infections (42, 43). Furthermore, the gut microbiota control immune regulation and are involved in the development and maturation of the human immune system in humanized mice (44). Reconstitution of the human immune system in immunodeficient mice alters the composition of intestinal flora.

The relative abundance and diversity of species were observed among three cohorts in the study, suggesting that differences in immune systems lead to differences in gut microbiota between NCG mice and huNCG mic. Firmicutes and Bacteroidetes are the predominant bacterial phyla colonizing the human gut (45). The abundance of Firmicutes and Bacteroidetes at the phylum level was increased in huNCG mice compared with NCG mice. This suggests that the gut microbiota can be impacted by the immune system reconstitution process in humanized mice.

The immune system influences colonization by exogenous microorganisms. However, it was unclear whether the humanized mice would more accept the exogenous colonizing community of humans. The distinct bacterial colonization patterns have been reported in C57BL/6 J mice and immunodeficient NSG mice following human FMT (38). The C57BL/6 J mice, NCG mice, and huNCG mice underwent human FMT. The presence of native gut microbiota resulted in resistance to colonization by human fecal microbiota (38). To eliminate the impact of this colonization resistance on the gut microbiota, antibiotic pretreatment was applied before human FMT. The composition and function of the gut microbiota were similar between huNCG mice and human donors after human FMT compared with C57BL/6 J mice and NCG mice, suggesting a similar immune microenvironment for same selected species, more easily colonized similar exogenous colonizers. The strain-level selection on exogenous species is exerted by the immune microenvironment.

The *Bacteroides plebeius*, *Bacteroides finegoldii*, *Klebsiella pneumoniae*, and *Klebsiella variicola strains* exhibited higher abundance and colonization stability in huNCG mice compared with NCG mice. Phylogenetic analysis showed that the four strains indeed had increase abundance. The increased abundance of *Bacteroides plebeius* can restore gut microbiota dysbiosis and optimize commensal bacteria (46). However, we found that humanized mice are highly susceptible to infection by novel human pathogenic bacteria, including *Bacteroides finegoldii*, *Klebsiella pneumoniae*, *Klebsiella variicola,* and *Escherichia coli* (47–49), and *Escherichia coli* significantly increased in all the mice after FMT, which can produce bacteriocins, which are short and toxic peptides that inhibit the colonization and growth of other species in the gut (50, 51). These factors can help explain the prevalence of toxic bacteria over beneficial bacteria.

Although most bacteria can efficiently colonize in the humanized mice after FMT, current FMT experiments cannot fully simulate the gut microbiota of the donor (52–56). *Megamonas* is a unique flora that is significantly positively associated with inflammatory diseases and metabolic disorders in human donors (57, 58), but it is not found in all colonized groups. This may be due to the fact that huNCG mice lack macrophages and cannot completely simulate the human immune system, thus limiting part of the effects of the immune system on human bacterial colonization.

In summary, differences in the immune microenvironment lead to differences in intestinal flora. The colonization ability of bacterial flora is also closely related to the immune microenvironment. The humanized immune microenvironment phenotype would more colonize the exogenous colonizing community, and pathogenic bacteria are more dominant. This provides a foundation for investigating the pathogenesis of various immune-related diseases, such as inflammatory bowel disease, stroke, and autoimmune diseases. However, a major limitation of this study was the use of 16 S r RN A gene sequencing rather than metagenomic sequencing, which limits the interpretation of the data at the species level and in terms of functional analysis. The study also provides initial insights to clarify which microbiota can be researched in the humanized mouse model. The future studies may use a gnotobiotic humanized mouse model to assess the relationship between human disease and microbiota.

## MATERIALS AND METHODS

### Study design

C57BL/6 J mice and NOD/ShiLtJGpt-*Prkdc^em26Cd52^Il2rg*^em26Cd22^/Gpt (NCG) mice (male, 12-week-old, *n* = 6) were acquired from GemPharmatech LLC. Six 12-week-old male NCG mice were constructed by reconstructing the human immune system through transplantation of human hematopoietic stem cells (hu-HSCs). Mice were divided into three groups based on the immune system: (1) C57BL/6 J mice with an intact immune system; (2) NCG mice with a defective immune systems; (3) humanized NCG mice with a human immune system. Fecal samples were collected before antibiotic administration (D0) and then again after 3 consecutive days of FMT (D9), followed by additional fecal collections in the subsequent 3 weeks (D16, D23, and D30). The mice were housed in a facility of Xuzhou Medical University that was free from pathogens. The mice were maintained in a 12-h cycle of light and darkness, with a constant temperature and humidity of 23 ± 1°C and 55%–65%. They were given food of the same quality and allowed unlimited access to water. Animal experimental procedures were conducted following the ethical guidelines established by the Xuzhou Medical University (approval number XZYK20230302).

### CD34^+^ cell isolation

The EasySep kit procedure for selecting human cord blood CD34^+^ cells (Stemcell Technologies, Canada) was used to acquire cord blood CD34^+^ cells. Briefly, cord blood was first pre-enriched and then diluted 1:1 with PBS. The diluted cord blood was layered on an equal volume of Ficoll solution and centrifuged to separate mononuclear cells using a density gradient. CD34^+^ cells were then obtained by magnetic bead selection.

### Humanization procedure

Flow cytometry was employed to stain the acquired hCD34^+^ cells in order to guarantee the quality of isolated CD34^+^ cells. Only hCD34^+^ cells with a purity greater than 95% were used for the construction of humanized mice. NCG mouse pups were irradiated with 120 cGy using the X-RAD 225XL irradiation system (Precision X-ray irradiation, USA). A total of 100,000 hCD34^+^ cells were promptly injected into the liver of the mice after irradiation. Further research was conducted on humanized mice, which were defined as having above 25% of human CD45^+^ cells in the peripheral blood.

### Flow cytometry

Using a flow cytometer, the levels of human CD45^+^ cells and various subsets of human immune cells (including C D3^+^ T cells, C D19^+^ B cells, C D56^+^ NK cells, as well as other lineage-negative cells) in peripheral blood were assessed after 8 weeks of hCD34^+^ injection. All antibodies were purchased from BioLegend and were not cross-reactive in the respective channels (BioLegend, USA). Flow cytometry measurements were performed using a Becton Dickinson FACSCalibur flow cytometer (BD Bioscience, USA). The unprocessed data were examined utilizing FlowJo software.

### Human FMT in C57BL6/J, NCG, and huNCG mice

C57BL6/J, NCG, and huNCG mice were grouped (Fig. 1A). Broad-spectrum antibiotics were administered in drinking water to the mice for 1 week (1 mg/mL each of ampicillin, neomycin, metronidazole, and 0.35 mg/mL vancomycin). In order to provide a more comprehensive representation of the human fecal microbiota, three fecal samples from patients with stroke (obtained from Xuzhou Medical University Affiliated Hospital, China) were combined and dissolved in sterile *P* BS at a 100 mg/mL ratio. To ensure complete dissolution, the mixture was vortexed using a Vortex - Genie 2 tabletop vortexer (Scientific Industries, USA) at intensity 8 for thorough dissolution. The supernatant was collected after centrifugation at 4°C, 800 g for 3 minutes. The transplantation was performed using 10 µL/g of the supernatant via oral gavage for 3 consecutive days. Notably, due to changes in bacterial composition and activity over time during the experiment, a fresh fecal mixture was prepared using newly collected feces each day before gavage. The FMT experiment strictly follows the "Microbiology Experiment Protocol E-Book" (59). During the FMT process, sterile PBS was used to prepare a fecal bacterial suspension under anerobic conditions, and the transplantation process was controlled to be completed within 15 minutes.

### Sequencing of the 16S rRNA gene and DNA extraction

Fecal samples were collected in tubes and immediately frozen in liquid nitrogen to preserve microbial DNA. Bacterial genomic DNA was extracted using the Qiagen QIAamp Fast DNA Stool Mini Kit. Specifically, the polymerase chain reaction (PCR) was used to amplify the 16S rRNA V3–V4 region. The amplified DNA fragments were then sequenced using Illumina’s MiSeq platform. The primers used for PCR amplification were 338F (5′-ACTCCTACGGAGGCAGCAG-3′) and 806R (5′-GGACTACHVGGGGTWTCTAAT-3′).

### Phylogenetic tree construction

Multiple sequence comparisons and phylogenetic trees were constructed for each locus using MEGA11 software (60). A phylogenetic tree was constructed by applying the maximum likelihood method (61), and the distance matrix of dissimilarity values was calculated using the general time reversible model (62). A total of 1,000 bootstrap analyses were also performed in the ML analysis.

### Bioinformatic analysis

The 16 S rRNA sequencing data underwent preprocessing using QIIME2 software (version 2021.11) (63). Quality filtering was performed, and a feature table was generated using the DADA2 tool. Using the vsearch plugin, operational taxonomic units (OTUs) had been classified at a 97% sequence similarity threshold, and taxonomic classification was performed based on the Silva (SSU138) 16S rRNA database (version 13. 8). To ensure data reliability, OTUs that accounted for less than 0.005% of the total sequences were excluded. The generated data set had an average of 35,393 reads per sample (minimum: 25,443 reads; maximum: 45,161 reads). Using the FastTree plugin, a phylogenetic tree was created after the sequences were aligned using MAFFT. With a sampling depth of 25,443, q2-diversity was utilized to analyze the diversity of the gut microbiome. Using PICRUSt and 16S rRNA sequences, the gut microbiome’s function was calculated, as previously described (64).

The Kruskal–Wallis test was employed to compare α-diversity indices, and pairwise comparisons were conducted using the Mann–Whitney U test. The unweighted UniFrac distance metric was used for β-diversity analysis to analyze the variation in the microbial community structure among samples, and the results were visualized using principal coordinate analysis. The Bonferroni method was used to correct the derived *P* values. A statistical technique known as analysis of similarity (ANOSIM) was used to determine the differences in unweighted UniFrac distances across groups. A statistical test known as one-way analysis of variance (ANOVA) was carried out to evaluate the relative levels of abundant microbial taxa at various taxonomic levels (phylum, family, and genus) between groups, and *post-hoc* tests were carried out to identify significant differences. Additionally, biomarkers for both abundant taxa and functional pathways were found using the linear discriminant analysis (LDA) effect size. A heatmap was generated using a software package called R. For predicting the abundance of functional categories, a technique called hylogenetic investigation of communities by reconstruction of unobserved states (PICRUSt) was utilized, based on operational taxonomic units (OTUs). Functional predictions were made using the orthology (KO) database from the Kyoto Encyclopedia of Genes and Genomes (KEGG).

### Statistical analysis

The data were presented using the mean ± SEM for *in vivo* and *in vitro* investigations, except those whose results were from 16S rRNA gene sequencing. Two-tailed Student’s t tests were used to compare the two groups. ANOVA was used to examine differences across different groups, and the Tukey multiple comparison test was used for *post-hoc* analysis. The cutoff for statistical significance was *P* < 0.05.

## Data Availability

The original contributions presented in the study are included in the article; further inquiries can be directed to the corresponding authors. The original data can be found from https://figshare.com/s/c7e69ffa4ac9f21fbb29.

## SUPPLEMENTAL MATERIAL

Supplemental material spectrum.00436-24-s0001.docx Fig. S1 and S2; Table S1.

Graphical abstract spectrum.00436-24-s0002.tif

ASM does not own the copyrights to Supplemental Material that may be linked to, or accessed through, an article. The authors have granted ASM a non-exclusive, world-wide license to publish the Supplemental Material files. Please contact the corresponding author directly for reuse.
