# Supplementary material for: A novel clinically relevant human fecal microbial transplantation model in humanized mice
Source: Microbiol Spectr. 2024 Aug 20;12(10):e00436-24. doi: 10.1128/spectrum.00436-24 (PMC11448399; doi:10.1128/spectrum.00436-24)
Supplement: Supplemental material — Fig. S1 and S2; Table S1. [file spectrum.00436-24-s0001.docx]

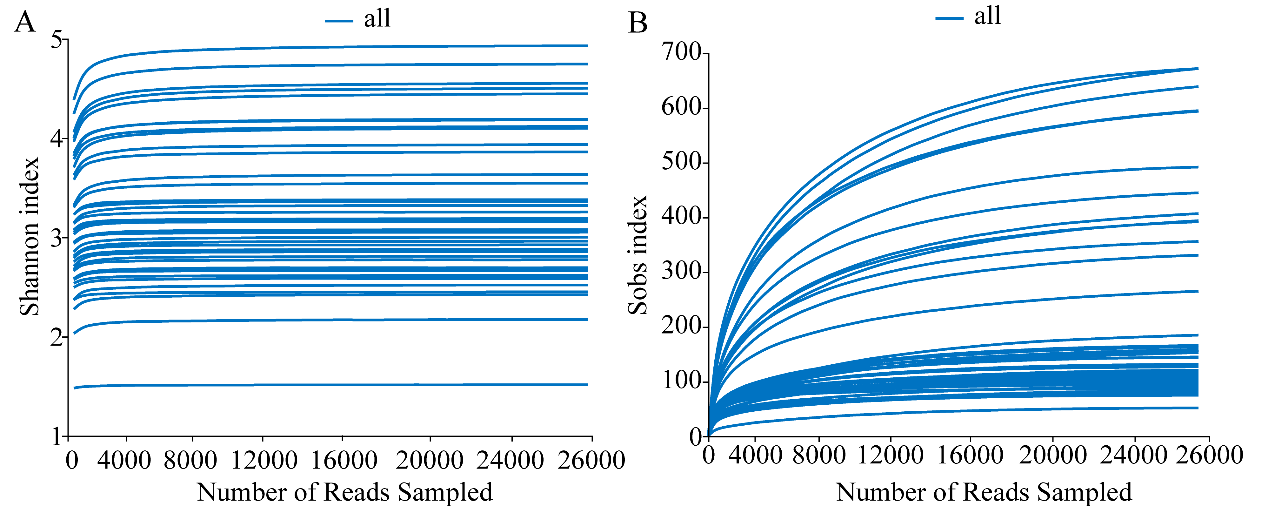


**FigureS1. Sequencing sample depth for dilution curve analysis.** Dilution curves for all samples constructed from (A) Shannon index and (B) Sobs index.


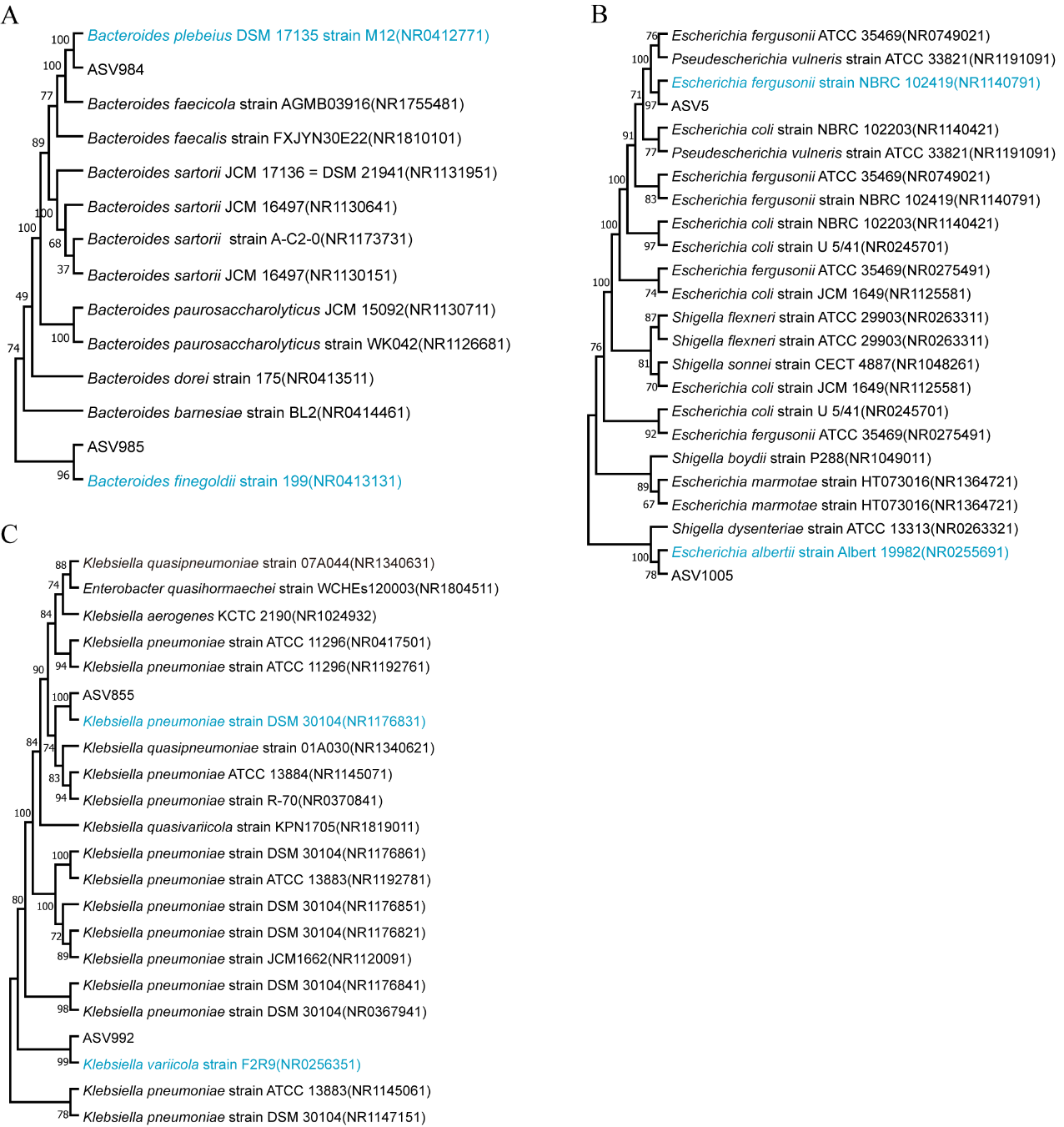


**FigureS2. Phylogenetic analysis of the amplicon sequence variants (ASVs) in huNCG mice.** Phylogenetic consensus trees of **(A)** *Bacteroides*, **(B)** *Escherichia*, and **(C)** *Klebsiella* were reconstructed from the 16S rRNA gene sequences using the Maximum Likelihood algorithm based on the General Time Reversible model, indicating the taxonomic position of ASVs. The percentage of replicate trees in which the associated taxa clustered together in the bootstrap test of 1000 replications are shown. Type strains are marked in blue font.

| ASV number | ASV sequences |
| --- | --- |
| ASV5 | TGGGGAATATTGCACAATGGGCGCAAGCCTGATGCAGCCATGCCGCGTGTATGAAGAAGGCCTTCGGGTTGTAAAGTACTTTCAGCGGGGAGGAAGGGAGTAAAGTTAATACCTTTGCTCATTGACGTTACCCGCAGAAGAAGCACCGGCTAACTCCGTGCCAGCAGCCGCGGTAATACGGAGGGTGCAAGCGTTAATCGGAATTACTGGGCGTAAAGCGCACGCAGGCGGTTTGTTAAGTCAGATGTGAAATCCCCGGGCTCAACCTGGGAACTGCATCTGATACTGGCAAGCTTGAGTCTCGTAGAGGGGGGTAGAATTCCAGGTGTAGCGGTGAAATGCGTAGAGATCTGGAGGAATACCGGTGGCGAAGGCGGCCCCCTGGACGAAGACTGACGCTCAGGTGCGAAAGCGTGGGGAGCAAACAGG |
| ASV855 | TGGGGAATATTGCACAATGGGCGCAAGCCTGATGCAGCCATGCCGCGTGTGTGAAGAAGGCCTTCGGGTTGTAAAGCACTTTCAGCGGGGAGGAAGGCGTTAAGGTTAATAACCTTGGCGATTGACGTTACCCGCAGAAGAAGCACCGGCTAACTCCGTGCCAGCAGCCGCGGTAATACGGAGGGTGCAAGCGTTAATCGGAATTACTGGGCGTAAAGCGCACGCAGGCGGTCTGTCAAGTCGGATGTGAAATCCCCGGGCTCAACCTGGGAACTGCATTCGAAACTGGCAGGCTAGAGTCTTGTAGAGGGGGGTAGAATTCCAGGTGTAGCGGTGAAATGCGTAGAGATCTGGAGGAATACCGGTGGCGAAGGCGGCCCCCTGGACAAAGACTGACGCTCAGGTGCGAAAGCGTGGGGAGCAAACAGG |
| ASV984 | TGAGGAATATTGGTCAATGGACGAGAGTCTGAACCAGCCAAGTAGCGTGAAGGATGAAGGTCCTACGGATTGTAAACTTCTTTTATAAGGGAATAAACCCTCCCACGTGTGGGAGCTTGTATGTACCTTATGAATAAGCATCGGCTAACTCCGTGCCAGCAGCCGCGGTAATACGGAGGATGCGAGCGTTATCCGGATTTATTGGGTTTAAAGGGAGCGCAGACGGGTCGTTAAGTCAGCTGTGAAAGTTTGGGGCTCAACCTTAAAATTGCAGTTGATACTGGCGTCCTTGAGTGCGGTTGAGGTGTGCGGAATTCGTGGTGTAGCGGTGAAATGCTTAGATATCACGAAGAACTCCGATTGCGAAGGCAGCACACTAATCCGTAACTGACGTTCATGCTCGAAAGTGTGGGTATCAAACAGG |
| ASV985 | TGAGGAATATTGGTCAATGGGCGAGAGCCTGAACCAGCCAAGTAGCGTGAAGGATGACTGCCCTATGGGTTGTAAACTTCTTTTATAAAGGAATAAAGTCGGGTATGTATACCCGTTTGCATGTACTTTATGAATAAGGATCGGCTAACTCCGTGCCAGCAGCCGCGGTAATACGGAGGATCCGAGCGTTATCCGGATTTATTGGGTTTAAAGGGAGCGTAGATGGATGTTTAAGTCAGTTGTGAAAGTTTGCGGCTCAACCGTAAAATTGCAGTTGATACTGGATATCTTGAGTGCAGTTGAGGCAGGCGGAATTCGTGGTGTAGCGGTGAAATGCTTAGATATCACGAAGAACTCCGATTGCGAAGGCAGCCTGCTAAGCTGCAACTGACATTGAGGCTCGAAAGTGTGGGTATCAAACAGG |
| ASV992 | TGGGGAATATTGCACAATGGGCGCAAGCCTGATGCAGCCATGCCGCGTGTGTGAAGAAGGCCTTCGGGTTGTAAAGCACTTTCAGCGGGGAGGAAGGCGGTGAGGTTAATAACCTCACCGATTGACGTTACCCGCAGAAGAAGCACCGGCTAACTCCGTGCCAGCAGCCGCGGTAATACGGAGGGTGCAAGCGTTAATCGGAATTACTGGGCGTAAAGCGCACGCAGGCGGTCTGTCAAGTCGGATGTGAAATCCCCGGGCTCAACCTGGGAACTGCATTCGAAACTGGCAGGCTAGAGTCTTGTAGAGGGGGGTAGAATTCCAGGTGTAGCGGTGAAATGCGTAGAGATCTGGAGGAATACCGGTGGCGAAGGCGGCCCCCTGGACAAAGACTGACGCTCAGGTGCGAAAGCGTGGGGAGCAAACAGG |
| ASV1005 | TGGGGAATATTGCACAATGGGCGCAAGCCTGATGCAGCCATGCCGCGTGTATGAAGAAGGCCTTCGGGTTGTAAAGTACTTTCAGCGGGGAGGAAGGGAGTAAAGTTAATACCTTTACTCATTGACGTTACCCGCAGAAGAAGCACCGGCTAACTCCGTGCCAGCAGCCGCGGTAATACGGAGGGTGCAAGCGTTAATCGGAATTACTGGGCGTAAAGCGCACGCAGGCGGTTTGTTAAGTCAGATGTGAAATCCCCGGGCTCAACCTGGGAACTGCATCTGATACTGGCAAGCTTGAGTCTCGTAGAGGGGGGTAGAATTCCAGGTGTAGCGGTGAAATGCGTAGAGATCTGGAGGAATACCGGTGGCGAAGGCGGCCCCCTGGACGAAGACTGACGCTCAGGTGCGAAAGCGTGGGGAGCAAACAGG |

**Table S1. the specific sequences of these 6 ASV sequences.**
